# A genetic risk score to predict treatment nonresponse in psychotic depression

**DOI:** 10.1038/s41398-024-02842-x

**Published:** 2024-03-02

**Authors:** Sophie E. ter Hark, Marieke J. H. Coenen, Cornelis F. Vos, Rob E. Aarnoutse, Willem A. Nolen, Tom K. Birkenhager, Walter W. van den Broek, Arnt F. A. Schellekens, Robbert-Jan Verkes, Joost G. E. Janzing

**Affiliations:** 1 Department of Psychiatry, Radboud University Medical Center, Nijmegen, The Netherlands; 2 Donders Institute for Brain, Cognition and Behaviour, Radboud University, Nijmegen, The Netherlands; 3 Department of Clinical Chemistry, Erasmus University Medical Center, Rotterdam, The Netherlands; 4 Department of Pharmacy, Radboud Institute for Health Sciences, Radboud University Medical Center, Nijmegen, The Netherlands; 5 Department of Psychiatry, University Medical Center Groningen, University of Groningen, Groningen, The Netherlands; 6 Department of Psychiatry, Erasmus University Medical Centre, Rotterdam, The Netherlands; 7 Nijmegen Institute for Scientist Practitioners in Addiction (NISPA), Radboud University, Nijmegen, The Netherlands

**Keywords:** Predictive markers, Depression

## Abstract

Psychotic depression is a severe and difficult-to-treat subtype of major depressive disorder for which higher rates of treatment-resistant depression were found. Studies have been performed aiming to predict treatment-resistant depression or treatment nonresponse. However, most of these studies excluded patients with psychotic depression. We created a genetic risk score (GRS) based on a large treatment-resistant depression genome-wide association study. We tested whether this GRS was associated with nonresponse, nonremission and the number of prior adequate antidepressant trials in patients with a psychotic depression. Using data from a randomized clinical trial with patients with a psychotic depression (*n* = 122), we created GRS deciles and calculated positive prediction values (PPV), negative predictive values (NPV) and odds ratios (OR). Nonresponse and nonremission were assessed after 7 weeks of treatment with venlafaxine, imipramine or venlafaxine plus quetiapine. The GRS was negatively correlated with treatment response (*r* = −0.32, *p* = 0.0023, *n* = 88) and remission (*r* = −0.31, *p* = 0.0037, *n* = 88), but was not correlated with the number of prior adequate antidepressant trials. For patients with a GRS in the top 10%, we observed a PPV of 100%, a NPV of 73.7% and an OR of 52.4 (*p* = 0.00072, *n* = 88) for nonresponse. For nonremission, a PPV of 100%, a NPV of 51.9% and an OR of 21.3 (*p* = 0.036, *n* = 88) was observed for patients with a GRS in the top 10%. Overall, an increased risk for nonresponse and nonremission was seen in patients with GRSs in the top 40%. Our results suggest that a treatment-resistant depression GRS is predictive of treatment nonresponse and nonremission in psychotic depression.

## Introduction

Psychotic depression is a severe and difficult-to-treat subtype of major depressive disorder (MDD) characterized by depression and the presence of delusions or hallucinations. It has a lifetime prevalence of 0.35% to 1% [1, 2]. Most guidelines recommend treatment with an antidepressant in combination with an antipsychotic [3, 4]. If treatment does not show any symptom reduction within four to six weeks, electroconvulsive therapy (ECT) should be considered [5].

With each treatment failure in MDD, the chance of response to the next antidepressant decreases and the risk of developing treatment-resistant depression increases [6]. There is currently no consensus on the definition of treatment-resistant depression. This is one reason for the variable estimations of prevalence, which vary between 7–22% [7, 8] and rates up to 35% in clinical trials [9]. The most common definition is failure to respond to two or more adequate treatment attempts [10, 11], although there are inconsistencies in the definition of what adequate treatment entails (i.e. what the required time and dose for an antidepressant trial is). A 2.2-fold higher risk of treatment-resistant depression (79.9% and 35.8% respectively, based on the number of antidepressant trials) has been observed in patients suffering from psychotic depression compared to patients with non-psychotic depression. [2].

To gain more insight into why and in whom treatment-resistant depression and antidepressant treatment failure occur, studies have linked a range of clinical and genetic characteristics to antidepressant nonresponse or treatment-resistant depression. Li et al. found clinical characteristics such as age, gender, depression severity and psychotic disorders to be associated with treatment-resistant depression [12]. Several studies using machine-learning approaches have been performed aiming to predict treatment-resistant depression or antidepressant treatment response using clinical characteristics [13–15]. These studies developed models with sufficient predictive ability based on large numbers of predictors with small effect sizes. These models seem promising in improving treatment outcomes, although replication studies are lacking which prevents clinical application. However, these machine-learning studies exclude patients with psychotic depression despite the fact that treatment-resistant depression is more prevalent in this population.

It has been frequently reported that genetic factors are involved in MDD, and depression-related outcomes [8, 16, 17]. Multiple genes and genetic variants have been linked to treatment response and treatment-resistant depression [18], as well as polygenic risk scores (PRSs) of psychiatric disorders (for review see [19]). For instance, a higher genetic load for both MDD and schizophrenia predicted antidepressant treatment nonresponse [20] and a higher genetic load for ADHD was seen in the treatment-resistant depression population [8]. In accordance with many other non-genetic prediction studies on treatment-resistant depression, the most severe populations including patients with psychotic depression were excluded. Despite several studies on treatment-resistant depression predictors [12], stratification tools [9, 13, 14] and staging models [21, 22], the risk of treatment-resistant depression or treatment nonresponse is not commonly assessed in clinical practice.

Identification of which psychotic depression patients are at risk for treatment nonresponse is important, as such information could be included in clinical decision-making (e.g. by more closely monitoring these patients or intensifying their treatment). In the current study, we evaluated the predictive ability of a treatment-resistant depression genetic risk score (GRS, a measure of an individual’s genetic liability to an outcome of interest [23]) to predict treatment nonresponse, nonremission and the association with the number of adequate trials with antidepressants in a sample of patients with psychotic depression [24]. Based on the high rates of treatment-resistant depression observed in psychotic depression [2], we hypothesized that patients with a high genetic load for treatment-resistant depression are less likely to show response or remission, and have used more antidepressants. Furthermore, we assessed to what extent including the GRS in a treatment nonresponse and nonremission prediction model would improve predictive power.

## Materials & methods

### Study sample

For this post hoc analysis, we used data from the Dutch University Depression Group (DUDG) study (ISRCTN36607067) [24]. This study was a multicenter double-blind randomized controlled trial with the aim to compare the efficacy of imipramine (plasma-concentrations 200–300 ug⁄l), venlafaxine (375 mg⁄day) and venlafaxine plus quetiapine (375 mg⁄day plus 600 mg/day) in psychotic depression. Study recruitment took place between June 2002 and June 2007. Study participants (*n* = 122) were hospitalized patients with a unipolar major depressive episode with psychotic features according to the DSM-IV-TR criteria and a Hamilton Rating Scale for Depression (HAM-D; 17-item version, score ≥18 [25]). Patients were aged 18–65 years and predominantly of European descent. Details are published elsewhere [24]. Before study initiation, patients were interviewed by a psychiatrist about their current depressive episode using the Structured Clinical Interview for DSM-IV Axis I Disorders (SCID-I). Data on previous drug treatments and ECT in the current episode were systematically collected using the Antidepressant Treatment History Form (ATHF [26]). These data were complemented with information from medical referral letters and prescription data.

### Genotyping information

Samples were genotyped using the Illumina Infinium Global Screening Array-24 version 2.0, performed by the Human Genomics Facility at Erasmus MC, Rotterdam, The Netherlands. Quality control (QC) was conducted according to the RICOPILI pipeline, this included a Hardy-Weinberg equilibrium test (HWE > 1.0 × 10^−6^), Fhet +/−0.2, a minor allele frequency (MAF) > 0.05, a call rate>98%, a PI-HAT < 0.2 and a sex check [27]. Population outliers were removed from our dataset based on two principal components (*n* = 6). Genotype data were imputed (best guess) using the RICOPILI tool [27] and EUR 1000 Genomes Reference Panel V3. All single nucleotide polymorphisms (SNPs) with an INFO score>0.8 were selected after QC genotype data was available for 109 (89.3%) patients.

### Genetic risk score (GRS)

We used the results of a recent large genome-wide association study (GWAS) on treatment-resistant depression [8] to create a GRS. A GRS is a measure of an individual’s genetic liability to an outcome of interest, calculated by summing the product of the effect sizes for risk alleles by the number of copies of the risk alleles across all included SNPs [23]. In contrast to a PRS, which is an extension of a GRS that captures the additive effects of many, often hundreds of thousands of SNPs [23]. This GWAS was chosen based on the large sample size (*n* = 16,372), type of data used (i.e. prescription data), phenotype definition (i.e. treatment-resistant depression was defined as two antidepressant switches within one depressive episode (14 weeks) with a minimum duration use of 6 weeks) and extensive study method substantiation (e.g. MDD pleiotropy). This study included UK Biobank data (https://www.ukbiobank.ac.uk/) and selected patients with two depression codes. Patients with a psychotic, bipolar or substance abuse disorder diagnosis were excluded. GWAS summary statistics were publicly available. For the GRS, we included all SNPs (*n* = 52) falling below suggestive significance (*p* < 5.0 ×10^−6^) as none of the SNPs reached significance (p < 5.0 ×10^−8^).

The GRS was created using PLINK 1.9 [28]. The SNPs were extracted from our GWAS data with the – extract function. Due to differences in imputation reference panels (1000G versus Haplotype Reference Consortium and UK10K) 38 of the 52 SNPs were available [29]. These SNPs were pruned using the – indep-pairwise function (window size = 50, LD threshold r^2^ = 0.5) to obtain independent SNPs, which resulted in a GRS containing 14 independent SNPs (Supplementary Table 1). A weighted GRS was constructed (– score function) based on the number of effect alleles (i.e. 0, 1 or 2) and the effect size. The GRS was available for 109 patients, however two patients were excluded due to a discrepancy between the genetic and clinical identification number leaving 107 patients for analysis. A principal components analysis (*n* = 107) was performed (– pca function) to visualize population heterogeneity (Supplementary Fig. 2) and to correct for population stratification (Supplementary Table 3). Results were corrected for two and ten principal components.

### Outcome measures

The primary endpoint of this analysis was treatment nonresponse, which was defined as <50% decrease in HAM-D-17 score after 7 weeks of treatment compared to baseline (i.e. at treatment initiation). Secondary, we studied treatment nonremission and the number of adequate antidepressant trials. Nonremission was defined as having a HAM-D 17 score >7 after 7 weeks of treatment. The HAM-D 17 scores were rated by a clinician. To resemble Fabbri’s treatment-resistant depression phenotype [8], we counted the number of adequate antidepressant trials in the treatment history which was only available for the current episode. We included an antidepressant trial if it met the criteria for an adequate trial; a sufficient dose for ≥4 weeks and an ATHF reliability score of ≥3 (i.e. moderate) [26].

### Statistical analysis

All analyses were performed using PLINK 1.9 [28] and R version 3.6 [30]. Correlation analysis was used to assess the overlap between the GRS and the outcome measures. Pearson correlations were used for continuous outcome measures and point-biserial correlation tests were conducted for binary outcome measures using the ltm package in R [31]. We divided patients into top GRS deciles (i.e. 10, 20, 30, 40 and 50% versus the remaining %) to study the optimal cut-off for nonresponse and nonremission prediction [32] and we calculated positive prediction values (PPV), negative predictive values (NPV) and odds ratios (OR). Odds ratios were calculated using the formula (A/B)/(C/D), in which A represents the number of nonresponders in the top GRS group, and B the number of responders in the top GRS group. C represented the number of nonresponders in the reference group and D the number of responders in the reference group. P-values reflect the significance levels of the odds ratios and were calculated as described by Sheskin [33]. Patients with missing data were not included in the analyses.

Logistic and linear regression analyses were used to evaluate the predictive ability of treatment nonresponse, nonremission and number of adequate antidepressant trials correcting for possible confounders. Regression analyses were performed using the lm() and glm() function in R.

The predictive ability was also presented in an area under the curve (AUC) from a Receiver Operating Characteristic (ROC) curve using the pROC package in R [34]. An area under the ROC curve (AUC) value of 0.5 indicates that a test has no discriminatory capacity and an AUC of 1.0 indicates perfect discriminatory capacity. For screening purposes, an AUC of 0.7 or higher is usually considered sufficient [35]. The optimal sensitivity and specificity with positive and negative predictive values from the ROC curves were calculated using the Youden method [36]. All regression models included the variables age, gender and depression severity (according to the Clinical Global Impression scale [37]) as these traits were significantly associated with treatment-resistant depression [12]. Logistic models assessing treatment nonresponse or nonremission also included the treatment arm (venlafaxine plus quetiapine), as the venlafaxine plus quetiapine treatment arm showed significantly more improvement than venlafaxine monotherapy [24]. We adjusted the p-value for the number of independent tests, this resulted in an alpha level of 0.025 for the regression analyses. Treatment nonresponse and treatment remission were not counted as independent outcome measures.

## Results

The patient characteristics are presented in Table 1. There were no significant differences between the whole sample (*n* = 122) and the sample for which genetic (*n* = 107) information was available. Of the 107 patients, 19 (17.8%) dropped out before study completion. As a result, the analyses using treatment nonresponse and nonremission included 88 patients and analyses on a number of adequate antidepressant trials included 107 patients. Of these 88 patients, 56 (63.6%) showed response and 38 (43.2%) achieved remission after seven weeks.

**Table 1:** Characteristics of the study sample (*n* = 107).

	Study sample (*n* = 107)
Gender	
Male	56 (52.3%)
Female	51 (47.7%)
Age (years)	51.0 (±10.4)
Number of depressive episodes	1.87 (±1.02)
Duration of current episode (weeks)	14 (2–676)
Number of adequate antidepressant trials of the current episode	
0	81 (75.7%)
1	21 (19.7%)
2	4 (3.7%)
3	0 (0.0%)
4	1 (0.9%)
Baseline HAM-D17 score	31.8 (±5.2)
Response	
Yes	56 (52.3%)
No	32 (29.9%)
Missing	19 (17.8%)
Remission	
Yes	38 (35.5%)
No	50 (46.7%)
Missing	19 (17.8%)

Values are presented as mean (±standard deviation (sd)), median (range) or number (%).

### GRS, treatment nonresponse and nonremission

The GRS consisted of 14 SNPs (Supplementary Table 1) and was normally distributed (Supplementary Fig. 1), which implies that population stratification is absent [38]. The median of GRS was 6.4 × 10^−3^ with a range of 1.1 × 10^−4^ to 1.3 × 10^−2^. No population heterogeneity was detected in the scatterplots showing the first three principal components. These were plotted for the total population (*n* = 107) and for patients showing response (*n* = 56) and patients showing nonresponse (*n* = 32) separately (Supplementary Fig. 2). The GRS was negatively correlated with both response (*r* = −0.32, 95% confidence interval (CI)(−0.50;−0.12), *n* = 88, *p* = 0.0023) and remission (*r* = −0.31, 95% CI(−0.48;−0.10), *n* = 88, *p* = 0.0037), but was not correlated with the number of adequate antidepressant trials (*r* = −0.08, 95% CI(−0.11;0.27), *n* = 107, *p* > 0.05). This means that patients with a high GRS for treatment-resistant depression were less likely to respond and to reach remission, however these are crude correlations not adjusted for covariates.

### Odds ratios

We studied associations by comparing the unadjusted odds ratios(OR) and the predictive values (i.e. PPV, NPV and accuracy) for treatment nonresponse and nonremission between the GRS group and the reference group (Table 2). Overall, the top 10% GRS showed the best predictive values for nonresponse, and these values were lower for nonremission. The PPVs of the top 10% GRS group were 100% for both treatment nonresponse and nonremission, this was combined with a NPV of 73.7% and 51.9%, respectively. This translated into an OR of 52.4 (*p* = 0.00072) for treatment nonresponse and an OR of 21.3 (*p* = 0.036) for nonremission. The accuracy for treatment nonresponse prediction decreased when lower deciles of the GRS were included, however the OR remained significant for patients with GRSs in the top 40% (OR = 4.4, *p* = 0.0018). Results for treatment nonremission were similar, except for the top 20% GRS group for which the p-value just fell below significance. The NPV values for nonremission and the PPV for nonresponse for the top 50% did not predict well, it was comparable to an at random prediction.

**Table 2:** PPVs, NPVs, accuracies and ORs for nonresponse and nonremission per GRS group.

Endpoint	GRS group	Reference group	PPV	NPV	Accuracy	ORs	95% CI	*p* value
Nonresponse	top 10%	0–90%	100.0%	73.7%	76.4%	52.37	2.91–940.75	**0.00072**
	top 20%	0–80%	66.7%	71.4%	70.5%	5.00	1.65–15.15	**0.0044**
	top 30%	0–70%	60.0%	73.0%	69.3%	4.06	1.53–10.76	**0.0048**
	top 40%	0–60%	57.6%	76.4%	69.3%	4.38	1.73–11.10	**0.0018**
	top 50%	0–50%	45.5%	72.7%	59.1%	2.22	0.91–5.41	0.079
Nonremission	top 10%	0–90%	100.0%	51.9%	57.8%	21.34	1.22–374.54	**0.036**
	top 20%	0–80%	77.8%	51.4%	56.8%	3.31	0.99–11.04	0.052
	top 30%	0–70%	76.0%	49.2%	56.8%	3.27	1.15–9.27	**0.026**
	top 40%	0–60%	75.8%	45.5%	56.8%	3.75	1.44–9.76	**0.0068**
	top 50%	0–50%	63.6%	50.0%	56.8%	1.75	0.75–4.10	0.20

Significant *p*-values are depicted in bold. Nonresponse was defined as <50% decrease in HAM-D 17 score, nonremission was defined as HAM-D-17 score >7. The results do not take possible confounders into account, adjusted effect sizes can be found in Supplementary Table 4.

### Regression models

Logistic and linear regression models were used to evaluate whether the GRS improved the coefficient of determination (R^2^) of treatment nonresponse and nonremission to a basic model (including age, gender, treatment arm and depression severity). Both models improved substantially when the GRS was added (Table 3). The R^2^ of the model for treatment nonresponse increased from 0.06 to 0.19. For treatment nonremission, the R^2^ improved from 0.02 to 0.13. The model for the number of adequate antidepressant trials did not improve when the GRS was added. Also, we included two and ten principal components in our analyses to correct for population stratification, these covariates did not change our results. The addition of two and ten principal components to our nonresponse model increased the adjusted R^2^ from 0.19 to 0.20 and 0.21 respectively (Supplementary Table 3). In the remission model, the adjusted R^2^ increased from 0.13 to 0.18 and 0.15 when including two and ten principal components respectively (Supplementary Table 3). Effect sizes and corresponding p-values per variable are presented in Supplementary Table 4.

**Table 3:** Logistic and linear regression models of treatment nonresponse, nonremission and number of adequate antidepressant trials.

Endpoint	Included variables	Adjusted R^2^	*P* value^a^
Nonresponse	Age, gender, treatment arm and depression severity	0.058	0.060
	Age, gender, treatment arm, depression severity and GRS	0.19	**0.00047**
Nonremission	Age, gender, treatment arm and depression severity	0.021	0.19
	Age, gender, treatment arm, depression severity and GRS	0.13	**0.0081**
Number of adequate antidepressant trials	Age, gender and depression severity	−0.011	0.61
	Age, gender, depression severity and GRS	−0.012	0.59

^a^Level of significance of the total regression model (Significant *p*-values (*p* < 0.025) are depicted in bold).

### Prediction of treatment nonresponse, nonremission and resistance

**Fig. 1:**
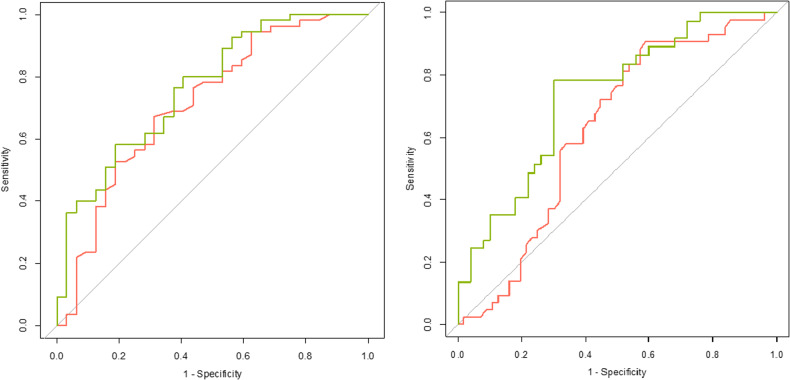
ROC curves of prediction on treatment nonresponse (left) and treatment nonremission (right). The green line shows the prediction by age, gender, treatment arm, depression severity and GRS. The red line shows the prediction without the GRS (age, gender, treatment arm and depression severity). Specificity on the x-axis and sensitivity on the y-axis.

The prediction of nonresponse and nonremission by the GRS was evaluated through the AUC of a ROC curve (Fig. 1). Both AUCs increased when adding the GRS to the basic models. In treatment nonresponse, the AUC increased by 7% (from 0.715 95% CI(0.598–0.832) to 0.765 95% CI(0.661–0.865)) and both values fell into the acceptable predictive value category [35]. An increase of 17% of the AUC was observed (from 0.626 95% CI(0.515–0.737) to 0.734 95% CI(0.629–0.8391)) for nonremission by GRS addition, the category changed from poor to acceptable. In line with previous results, the AUC of the number of adequate antidepressant trials did not improve after addition of the GRS and remained in the poor prediction category (i.e. AUC < 0.60, data not shown).

## Discussion

In this study, we evaluated the predictive ability of a treatment-resistant depression GRS from a nonpsychotic MDD cohort (originated from the UK biobank) on treatment response, remission and the number of adequate antidepressant trials in a clinical psychotic depression sample. As hypothesized, we found that patients with a high treatment-resistant depression GRS were less likely to show response and remission. We show that the risk of nonresponse and nonremission is the highest (PPV = 100%) in patients within the top 10% of the treatment-resistant depression GRS and that patients with a GRS in the top 40% are more likely to show nonresponse and nonremission compared to patients with lower GRSs. Secondly, we showed that adding a treatment-resistant depression GRS to a prediction model consisting of basic variables improves the prediction of treatment nonresponse and nonremission in patients with psychotic depression, but not the number of adequate antidepressant trials.

To our best knowledge, this is the first study to examine the predictive ability of a treatment-resistant depression GRS in treatment nonresponse and nonremission in patients with psychotic depression. Frequently used approaches in treatment prediction studies are machine-learning [9, 14] and PRSs analyses (for review see [19]), which study large amount of predictors. Both machine-learning and PRS studies show promising results, however, they both require large amounts of data and are computationally burdensome. Moreover, none of the predictors analyzed in these studies equals the predictive ability of this treatment-resistant depression GRS. Despite that PRS studies offer in general a heightened predictive power over GRSs [39], it require a large sample size which makes this approach not suitable for psychotic depression.

The primary goal of prediction studies is translation to clinical practice. Before this treatment-resistant depression GRS can be implemented in clinical practice, some steps will have to be taken such as the establishment of the clinical validity and clinical utility. We studied the clinical validity (i.e. the ability of the test to detect or predict the clinical disorder or phenotype associated with the genotype [40]). We showed that the PPV of treatment nonresponse and nonremission was 100% for patients (*n* = 9) with a GRS in the top 10%, meaning that the prediction of nonresponse and nonremission was correct for all patients in this group. The NPVs were smaller; 73.7% for treatment nonresponse and 51.9% for nonremission. In addition, we showed that the GRS has an added value in patients with GRSs in the top 40%. Although the accuracy of the prediction is lower in that group, it would mean that a larger number of patients could be guided by the GRS. Currently, there are no established predictive cut-off values for GRSs. For antidepressant response predictions, the highest cut-off values (top 10–20%) are of special interest as these are associated with high values for PPV and specificity. However, these values are counterbalanced by a moderate sensitivity (some patients will be labelled incorrectly as not treatment-resistant). Depending on the therapeutic consequences and the availability of other predictive variables, the GRS cut-off values can be lowered resulting in an increase in sensitivity but at the expense of specificity. When taking into account that there are currently no tools used in MDD to predict treatment nonresponse or nonremission, a prediction by a genetic test could potentially be helpful. For now, the clinical validity is not yet established, first, a replication study should be conducted. Regarding the clinical utility of the GRS, it should be studied by looking at its usefulness in the clinic and its ability to change clinical endpoints [40].

The number of adequate antidepressant trials in our study did not show an association with the treatment-resistant depression GRS. Although, our phenotype was created to resemble the treatment-resistant depression phenotype by Fabbri et al. the absence of an association could be well explained by several differences. First, we only had information on the current depressive episode while Fabbri et al. had access to the complete medical history of patients. This resulted in a much lower treatment-resistant depression (i.e. two or more adequate antidepressant trials) rate in our sample (4.7% compared to 13.2% (Fabbri et al.)). Subsequently, we decided not to include the dichotomous measure of treatment-resistant depression as the number of patients that were classified for treatment-resistant depression was low (*n* = 5). Secondly, the definitions of adequate treatment differed. We used the ATHF in our study which rates the adequacy of antidepressant trials on duration (four weeks), dose and reliability (including compliance and treatment effect). Antidepressants in the treatment-resistant depression phenotype used by Fabbri et al. were included with a minimum use of six weeks and had no requirements regarding dose or compliance.

### Strengths & limitations

Our study has several strengths. We focused on psychotic depression and used a homogeneous sample of patients. A large number of studies on MDD are available but only a few focus on psychotic depression. Also, patients with psychotic depression are frequently excluded in MDD studies. However, studies focusing on psychotic depression are essential to improve treatment outcomes of this difficult-to-treat MDD subgroup. Further studies are necessary to investigate if our findings also apply to patients with other subtypes of MDD. Although the GRS was derived from a population cohort with a low risk of psychotic depression, it could be possible that the predictive ability is stronger in patients suffering from the most severe subtypes of depression, including psychotic depression. The main limitation of our study is that it is a post-hoc analysis which limits the external validity. Other limitations are mainly based on methodical issues. As described above, there were differences in study method between our study and the study which we extracted the GRS from. Also, the treatment-resistant depression definition we used in our study resulted in right-skewed data, as the majority of patients (*n* = 81, 75.7%) scored zero. Therefore, the lack of correlation is not unexpected. Furthermore, our study does not provide information on the association between the treatment-resistant depression GRS and the outcome of psychotic symptoms. Although a rating scale exists that integrates both psychotic and depressive symptoms (the Psychotic Depression Assessment Scale (PDAS; [41]), it was not used in our study. However, in psychotic depression ‘very similar’ treatment effect sizes have been measured for HAM-D-17 and the PDAS [42]. Also, some SNPs in the treatment-resistant depression GRS were not available in our sample. This was due to the different genotype methods, imputation and/or quality control steps. As a consequence, some information was lost and the predictive ability of the GRS was potentially underestimated. Lastly, our sample size is small compared to other genetic studies. Nonetheless, it is in the same range of the larger prospective studies in psychotic depression [43, 44].

## Conclusion

In conclusion, our study suggests that the treatment-resistant depression GRS might be valuable in predicting treatment nonresponse and nonremission in psychotic depression. As we performed a post-hoc and exploratory analysis, replication studies in other populations of depressed patients are necessary to corroborate our findings with the final aim to investigate the applicability in clinical practice.

### Supplementary information

Supplementary information

## Data Availability

Data are available from the authors upon reasonable request.
