# Correction: A genetic risk score to predict treatment nonresponse in psychotic depression

**DOI:** 10.1038/s41398-024-02912-0

**Published:** 2024-04-26

**Authors:** Sophie E. ter Hark, Marieke J. H. Coenen, Cornelis F. Vos, Rob E. Aarnoutse, Willem A. Nolen, Tom K. Birkenhager, Walter W. van den Broek, Arnt F. A. Schellekens, Robbert-Jan Verkes, Joost G. E. Janzing

**Affiliations:** 1 Department of Psychiatry, Radboud University Medical Center, Nijmegen, The Netherlands; 2 Donders Institute for Brain, Cognition and Behaviour, Radboud University, Nijmegen, The Netherlands; 3 Department of Clinical Chemistry, Erasmus University Medical Center, Rotterdam, The Netherlands; 4 Department of Pharmacy, Radboud Institute for Health Sciences, Radboud University Medical Center, Nijmegen, The Netherlands; 5 Department of Psychiatry, University Medical Center Groningen, University of Groningen, Groningen, The Netherlands; 6 Department of Psychiatry, Erasmus University Medical Centre, Rotterdam, The Netherlands; 7 Nijmegen Institute for Scientist Practitioners in Addiction (NISPA), Radboud University, Nijmegen, The Netherlands

**Keywords:** Predictive markers, Depression

Correction to: *Translational Psychiatry* 10.1038/s41398-024-02842-x, published online 2 March 2024

In this article the author’s name Tom K. Birkenhager was incorrectly written as Tom K. Birkerhäger.

The original article has been corrected.

