# Supplementary material for: A genetic risk score to predict treatment nonresponse in psychotic depression
Source: Transl Psychiatry. 2024 Mar 2;14:132. doi: 10.1038/s41398-024-02842-x (PMC10908776; doi:10.1038/s41398-024-02842-x)
Supplement: Supplementary file 1 — Supplementary information [file 41398_2024_2842_MOESM1_ESM.docx]

# Supplementary Information

Supplementary Table 1 SNPs used in GRS (n=14 SNPs)

| **Number** | **SNP rs number** | **Chromosome** | **Position (basepair) (GRCh37)** | **Effect allele** | **Effect size**  **(beta)** | **Number of SNPs removed by pruning** |
| --- | --- | --- | --- | --- | --- | --- |
| 1 | rs62153281 | 2 | 81737394 | T | 0.037 | 1 |
| 2 | rs186910289 | 2 | 137056724 | C | 0.038 | 1 |
| 3 | rs13078081 | 3 | 66852901 | C | 0.038 | 7 |
| 4 | rs115633075 | 3 | 137198107 | T | 0.040 | 7 |
| 5 | rs350802 | 5 | 92246357 | T | 0.035 | 1 |
| 6 | rs79834175 | 5 | 172419037 | T | 0.038 | 0 |
| 7 | rs56093609 | 6 | 21095911 | G | 0.036 | 0 |
| 8 | rs4960705 | 7 | 154455403 | G | 0.037 | 0 |
| 9 | rs116949009 | 8 | 140040373 | T | 0.038 | 0 |
| 10 | rs144495474 | 11 | 30232200 | C | 0.037 | 1 |
| 11 | rs116979514 | 11 | 45219710 | T | 0.035 | 0 |
| 12 | rs1493489 | 12 | 66944708 | G | -0.037 | 2 |
| 13 | rs7207933 | 17 | 79521239 | G | 0.037 | 1 |
| 14 | rs76686854 | 20 | 342463 | T | 0.037 | 3 |

Supplementary Figure 1 Density plot of GRS distribution in study sample (n=107). The GRS score on x-axis is expressed as standard deviations from the mean with 0 representing the mean. The y-axis represents the density.


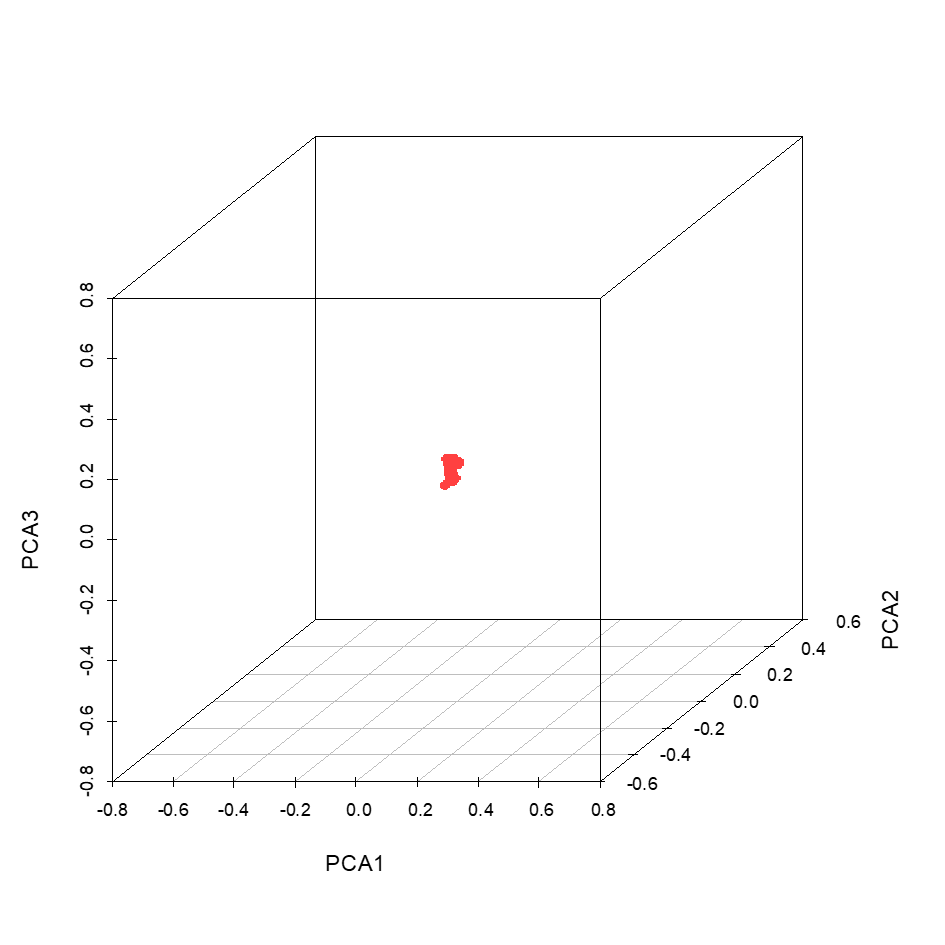

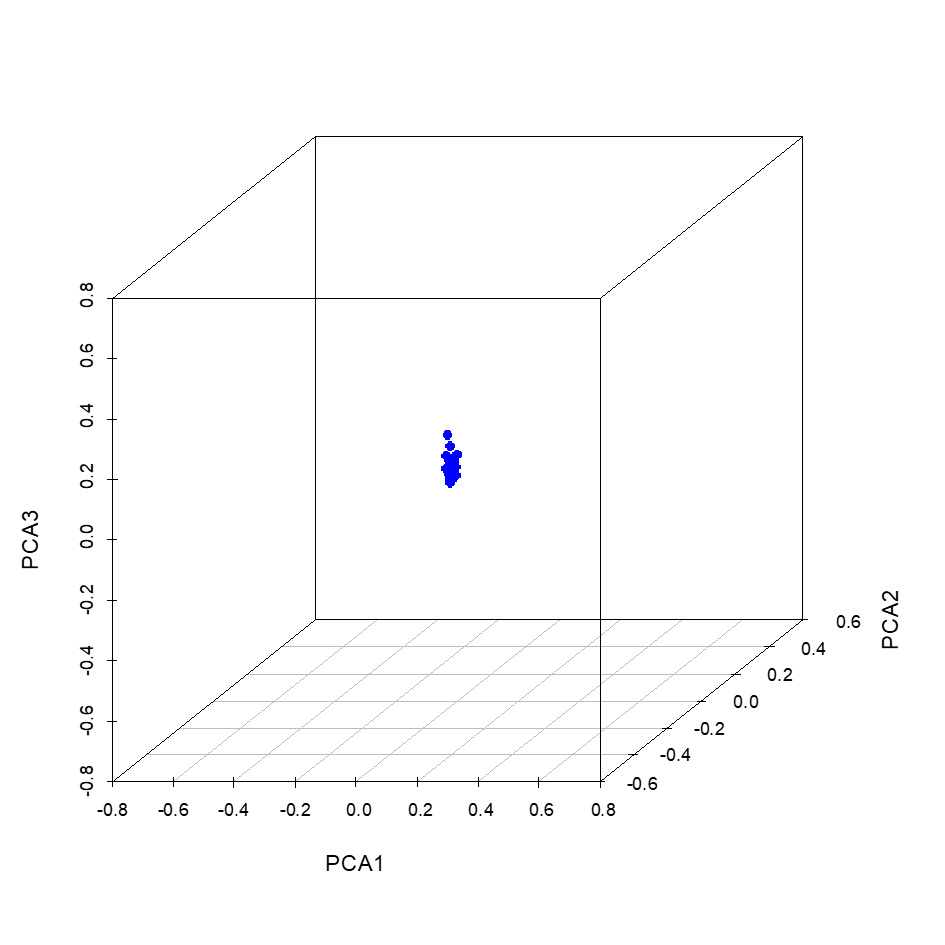
Supplementary Table 2 Sensitivity, specificity, nonremission and nonresponse rate per GRS decile

Supplementary Figure 2 3D scatterplots presenting the first three principal components from the study population A) presents the total study population (n=107), B presents the nonresponse population (n=32) and C presents the response population (n=56). On the x-axis the first principal component, on the z-axis the second principal component and on the y-axis the third principal component.

**C**

**B**

**A**

| Endpoint | GRS  group | Reference group | Sensitivity | Specificity | Response rate  (%) | Remission rate  (%) |
| --- | --- | --- | --- | --- | --- | --- |
| Nonresponse | top 10% | 0-90% | 31.0 | 100 | 0.0 |  |
|  | top 20% | 0-80% | 37.5 | 89.3 | 33.3 |  |
|  | top 30% | 0-70% | 46.9 | 82.1 | 40.0 |  |
|  | top 40% | 0-60% | 59.4 | 75.0 | 42.4 |  |
|  | top 50% | 0-50% | 62.5 | 57.1 | 54.5 |  |
| Nonremission | top 10% | 0-90% | 22.5 | 100.0 |  | 0.0 |
|  | top 20% | 0-80% | 29.2 | 90.0 |  | 22.2 |
|  | top 30% | 0-70% | 37.3 | 83.8 |  | 24.0 |
|  | top 40% | 0-60% | 45.5 | 75.8 |  | 24.3 |
|  | top 50% | 0-50% | 56.0 | 57.9 |  | 42.2 |

Supplementary Table 3 Logistic and linear regression models of treatment nonresponse, nonremission and number of adequate antidepressant trials including two and ten principal components as covariates. Significant p-values are depicted in bold.

| Endpoint | Included variables | Adjusted R^2^ | P-value |
| --- | --- | --- | --- |
| Nonresponse | Age, gender, treatment arm, depression severity, GRS and two principal components | 0.20 | **0.0010** |
|  | Age, gender, treatment arm, depression severity, GRS and ten principal components | 0.21 | **0.0040** |
| Nonremission | Age, gender, treatment arm and depression severity, GRS and two principal components | 0.18 | **0.0018** |
|  | Age, gender, treatment arm, depression severity, GRS and ten principal components | 0.15 | **0.023** |

Supplementary Table 4 Effect sizes and p-values per variable and outcome. Significant p-values are depicted in bold.

| Outcome | Covariates | Effect size | Standard error | P-value |
| --- | --- | --- | --- | --- |
| Nonresponse | TRD GRS | 408.26 | 146.16 | **0.0052** |
|  | age | -0.016 | 0.027 | 0.55 |
|  | gender | -0.62 | 0.53 | 0.24 |
|  | treatment arm | -1.47 | 0.58 | **0.011** |
|  | depression severity | 0.93 | 0.49 | 0.056 |
|  | 10 PCAs | 7.58 | 9.99 | 0.45 |
| Nonremission | TRD GRS | 438.90 | 148.60 | **0.0031** |
|  | age | -0.050 | 0.027 | 0.064 |
|  | gender | -0.73 | 0.50 | 0.14 |
|  | treatment arm | -0.89 | 0.51 | 0.082 |
|  | depression severity | 0.91 | 0.48 | 0.058 |
|  | 10 PCAs | -2.63 | 8.97 | 0.77 |
